# Human Dietary Exposure to Heavy Metals via Rice in Nepal

**DOI:** 10.3390/ijerph20054134

**Published:** 2023-02-25

**Authors:** Yuxiao Shao, Xiaohang Xu, Le Wang, Jialiang Han, Hem Bahadur Katuwal, Shulin Jiao, Guangle Qiu

**Affiliations:** 1 School of Geography and Environmental Science, Guizhou Normal University, Guiyang 550025, China; 2 State Key Laboratory of Environmental Geochemistry, Institute of Geochemistry, Chinese Academy of Sciences, Guiyang 550081, China; 3 Key Laboratory of Karst Georesources and Environment, Ministry of Education, College of Resources and Environmental Engineering, Guizhou University, Guiyang 550025, China; 4 University of Chinese Academy of Sciences, Beijing 100049, China; 5 Center for Integrative Conservation, Xishuangbanna Tropical Botanical Garden, Chinese Academy of Sciences, Mengla 666303, China

**Keywords:** heavy metals, rice, Monte Carlo simulation, estimated daily intake, vulnerable population, Nepal

## Abstract

The effects of exposure to heavy metals (HMs) in rice on human health have become a global public health concern, particularly in countries where rice is consumed as a staple food. The concentrations of HMs, including cadmium (Cd), arsenic (As), lead (Pb), and copper (Cu), in commercial rice samples (n = 170) were analyzed to estimate the HM exposure of consumers in Nepal. The geometric mean concentrations of Cd, As, Pb, and Cu in commercial rice were 15.5 ± 16.0, 43.4 ± 19.6, 16.0 ± 14.0, and 1066 ± 1210 μg/kg, respectively, all below the maximum allowable concentrations (MACs) recommended by FAO/WHO. Generally, the average estimated daily intakes (EDIs) of Cd, As, Pb, and Cu were all below the oral reference doses (RfDs). However, young age groups were exposed to high levels of HMs, and the average EDI of As and the P99.9 EDIs of Cu and Cd were above the corresponding RfDs. The mean hazard index and total carcinogenic risk were 1.13 and 1.04 × 10^−3^ respectively, suggesting a potential non-carcinogenic risk (NCR) and a carcinogenic risk (CR) via rice consumption. Arsenic contributed the most strongly to NCR and Cd to CR. Overall, although the HM levels in rice were generally safe, the Nepalese population may be exposed to an elevated health risk from rice consumption.

## 1. Introduction

Food contamination with heavy metals (HMs), including cadmium (Cd), lead (Pb), arsenic (As), zinc (Zn), copper (Cu), and mercury (Hg), is common in contaminated agricultural regions [1] and a major concern worldwide [2,3]. Numerous studies have demonstrated that exposure to HMs can damage the organs of animals and humans, even at low levels [4,5,6,7]. Long-term exposure to HMs via the intake of contaminated food can cause many kinds of diseases, such as cancer, leukemia, genetic toxicity, and so on [8]. Around 600 million people are reportedly harmed by HM-contaminated food annually worldwide [9,10].

Rice is the staple food for over 50% of the world population, contributing over 70% of the food energy in developing Asian countries [11]. Heavy metals, particularly Cd, As, and Pb, are easily absorbed and accumulated by rice grains [12]. Numerous studies have demonstrated that rice is the prime source of HMs for humans in Asia [13,14,15]. The Fifth China Total Diet Study revealed that rice heavy metal concentrations in more than 20 regions in China exceeded the standard values, and there was a serious non-carcinogenic risk, especially in the southeast region [16]. Zeng reported that the concentrations of As and Cd in rice were up to 310 and 340 μg/kg, respectively, in Hunan Province, China [17]. In Ranau Valley, Sabah, Malaysia, the average concentrations of Cd, As, and Cu in rice grains were 540, 50, and 2610 μg/kg, respectively [18]. Kukusamude investigated the concentrations of Cd, As, Pb, and Cu in rice from Thailand and confirmed the risk to humans of HM exposure via rice [19]. Proshad reported that the average concentrations of Cr, As, Cd, and Pb in rice in Bangladesh were 16, 260, 2280, 1880, and 793 μg/kg, respectively, higher than the permissible limits of the World Health Organization (WHO) [20]. Soil contamination by arsenic, due to highly contaminated irrigation water, and its carryover effect to rice plants have been reported in Bangladesh and India [21]. Hence, exposure to HMs in rice and the potential health risks resulting from rice consumption have become increasing concerns [22,23].

Health risk assessment (HRA) is an effective deterministic evaluation model to recognize the impact of harmful elements on human health directly and quantitatively [23,24]. Generally, HRA is based on the concentration of the metal in the edible part of a food relative to a reference dose of the metal and the intake/body weight of the consumer [25]. Traditionally, HRA is usually calculated by some models with fixed parameters [26]. However, individual variations can bias the results of HRA due to individual variations [27]. Monte Carlo simulations (MCSs) can reduce the uncertainties in results by providing a health risk probability for HMs [28].

Nepal is a developing country, and its economy is also based on agriculture. Rice is the staple food in Nepal, and approximately 3.65 million tonnes are consumed annually [11]. With increasing anthropogenic activities, such as irrigation with wastewater, and the use of agricultural fertilizers, pesticides, and organic manure in farming, rice may become a primary dietary source of HMs, particularly Cd, Cu, and Pb, in the Bhaktapur district of Nepal [29]. Previous studies have reported that the mean concentrations of Cd and As in rice were 50 µg/kg (range: 13.9–80 µg/kg) and 180 µg/kg (range: 60–330 µg/kg), respectively, in this district [30,31,32], and that 25.7% of the tube wells in the Nawalparasi district of Terai, Nepal, were contaminated with As [33]. Meharg et al. even reported rice Cd concentrations from 12 countries, including 12 samples from a Nepal market with the mean 50 µg/kg [31]. The studies by Wang et al. indicated that exposure to total mercury and methylmercury in rice for pregnant woman resulted in losses of intelligence quotients of the newborn in Nepal [3]. Arsenic contamination of rice occurred through high-As irrigation water in Nawalparasi district, Nepal, and the As exposure level was up to 180 µg/kg [30]. Considering the high concentrations of HMs found in rice in these regions, determining the levels of HMs in rice across the whole of Nepal is essential, and the assessment of further HM exposure is necessary to guide future management. However, to the best of our knowledge, no nationwide study of the concentrations of HMs in rice in Nepal has been undertaken, let alone the HRA of HM exposure from rice consumption.

In this study, our work aimed to (i) determine the concentrations and distributions of Cd, As, Pb, and Cu in commercial rice in Nepal and (ii) assess the health risks posed by exposure to HMs via rice consumption to the Nepalese people.

## 2. Materials and Methods

### 2.1. Study Area

Nepal (27°42′ N, 85°19′ E) is a Himalayan and land-locked country in South Asia, which borders China to the north and India on the remaining three sides. The country covers a rectangular area of 147,181 km^2^ [34]. More than 86% of the country is mountainous, and only a small portion in the south is low-lying land, known as “Terai”. The climate in Nepal varies from subtropical to alpine within a short distance due to tremendous variation in topography and altitude (60 to 8848 m) [35]. Nepal is an agricultural country, in which two-thirds of the population depends on agriculture, and it contributes almost 34 percent of the GDP [36].

### 2.2. Sampling

**Figure 1:**
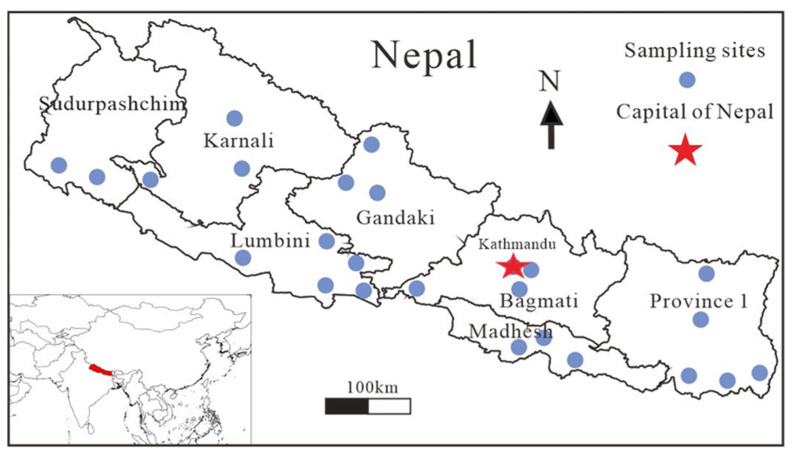
Sampling sites in different provinces of Nepal.

In September 2019, 170 rice samples were randomly collected from seven provinces of Nepal: Province 1 (n = 49), Madhesh (n = 20), Bagamti (n = 27), Gandaki (n = 13), Lumbini (n = 35), Karnali (n = 10), and Sudurpashchim (n = 16) (Figure 1). White rice samples that were intended for direct consumption by local residents were gathered from homes or markets. Each sample of no less than 100 g was collected in a polyethylene zip-locked bag. All the collected samples were taken to the laboratory for further processing.

### 2.3. Sample Preparation and Analysis

During sample processing, ~30 g of the sample was thoroughly washed with distilled deionized water (DDW). Washed samples were then dried using lyophilizer (FDU-2110, EYELA, Japan). The dried samples were then milled into a powder (about 80 meshes) and packed into sealed sample bags for further analysis. In the digesting process of the sample, approximately 0.2 g of the sample was digested with 5 mL of ultra-pure HNO_3_ in a Teflon digestion vessel and heated at 150 °C for 48 h in an oven. After cooling, 1 mL of 30% H_2_O_2_ was added and heated for 1 h at 90 °C on a heating plate. The temperature was gradually increased to 120 °C until all the solution had evaporated. Then, 1 mL HNO_3_ was added to the residual solution and heated until dry. After that, 3 mL of DDW and 2 mL of HNO_3_ were added to the remaining solution and heated at 150 °C for a further 6 h [37].

The digested solution was moved into a centrifuge tube and then diluted to a constant volume with DDW to maintain the acid concentration below 2%. Finally, the concentrations of the studied elements in the solution were determined using inductively coupled plasma–mass spectrometry (ICP-MS; NexION™ 300X, PerkinElmer, Waltham, MA, USA).

### 2.4. Quality Assurance and Quality Control (QA/QC)

The blanks, duplicate samples, and standard reference materials were used to assess the accuracy and precision of the method. The standard reference material (GBW 10020, citrus leaf; National Research Center for Standards, China) and a rhodium (Rh) internal standard were utilized for QA/QC. The recovery of Rh ranged from 86.5% to 116%. The method detection limits for As, Cd, Pb, and Cu were 2.4, 0.16, 0.8, and 6.4 μg/kg, respectively. Around 94.6–111.0% of the reference material was recovered, with a relative standard deviation of <5%, which indicate that the accuracy and precision of the analyses of all samples met the required procedure specifications.

### 2.5. Human Health Risk Assessment

#### 2.5.1. Estimated Daily Intakes (EDIs)

The EDIs of HMs depend on both the concentration of the HMs and the amount of rice consumed [38]. The EDI was calculated using following Equation (1):

(1) $$\mathrm{EDI}=\frac{\mathrm{CF}\;\times \;\mathrm{IR}\;\times \;\mathrm{EF}\;\times \;\mathrm{ED}}{\mathrm{BW}\times \mathrm{AT}}$$

where EDI (μg/kg/day) is the estimated daily intake. The description and values of all parameters in Equation (1) are listed in Table S1. The intake rate (IR) and body weight (BW) for the Nepalese in different regions were collected from former publications [39,40] (Table S2). 

#### 2.5.2. Non-Carcinogenic Risk Assessment

Non-cancer risk assessments were determined by calculating the respective hazard quotient (HQ) of contaminants. The HQ of each element was obtained by Equation (2) [41]:

(2) $$\mathrm{HQ}=\frac{\mathrm{EDI}}{\mathrm{RfD}}\;$$

where RfD (μg/kg/day) is the oral reference dose of the desired heavy metal, and the values for As, Cd, Pb, and Cu are 0.3, 1, 3.5, and 40 μg/kg/day, respectively [42]. HQ > 1 implies a potential risk of a non-carcinogenic effect [43].

The hazard index (HI) was used to assess the cumulative non-carcinogenic risk from multiple elements [44]. The HI was calculated using the following Equation (3):

(3) $$\mathrm{HI}=\sum_{n=1}^{i}{\mathrm{HQ}}_{n}\;$$

HI > 1 indicates a potential for adverse human health effect [45].

#### 2.5.3. Carcinogenic Risk Assessment

The carcinogenic risk (CR) and total carcinogenic risk (TCR) represent the probability of cancer caused by HMs in the rice consumed during a lifetime. CR and TCR were calculated using Equations (4) and (5), respectively:

(4) CR=EDI × SF

(5) $$\mathrm{TCR}={\mathrm{CR}}_{\mathrm{As}}+{\;\mathrm{CR}}_{\mathrm{Cd}}+{\;\mathrm{CR}}_{\mathrm{Pb}}$$

The SF in Equation (4) is the carcinogenicity slope factor [24]. As, Cd, and Pb are chemically carcinogenic, and their SF values are 1.5 × 10^−3^, 6.1 × 10^−3^, and 8.5 × 10^−3^ (mg/kg/day)^−1^, respectively [46]. TCR in Equation (5) is the total carcinogenic risk for As, Cd, and Pb.

According to USEPA’s guidelines for acceptable or tolerable CR and TCR, the range of acceptable values for CR or TCR is from 1.0 × 10^−6^ to 1.0 × 10^−4^. In general, if CR or TCR < 1.0 × 10^−6^, the cancer risk is considered negligible. However, if CR or TCR > 1.0 × 10^−4^, the cancer risk is considered unacceptable by most international regulatory agencies [47].

### 2.6. Statistical Analysis

The summary statistics were calculated using Microsoft Excel 2021. Statistical analyses were performed using SPSS 25 (IBM, Armonk, NY, USA), and Origin 2022 (OriginLab^®^ Corporation, Northampton, MA, USA) was used for drawing. The MCSs used for the uncertainty analyses were performed using Crystal Ball (Oracle, Redwood City, CA, USA). An MCS approach with 10,000 iterations was used to calculate dietary exposure to HMs. The different percentile levels from P50 to P99.9 were also determined using MCS. One-way analysis of variance (ANOVA) using Tukey’s post hoc test was used to determine the significance of the differences in HM concentrations among the different provinces, regions, and vulnerable populations, with a confidence level of 95%.

## 3. Results and Discussion

### 3.1. Concentrations of Cd, As, Pb, and Cu

**Figure 2:**
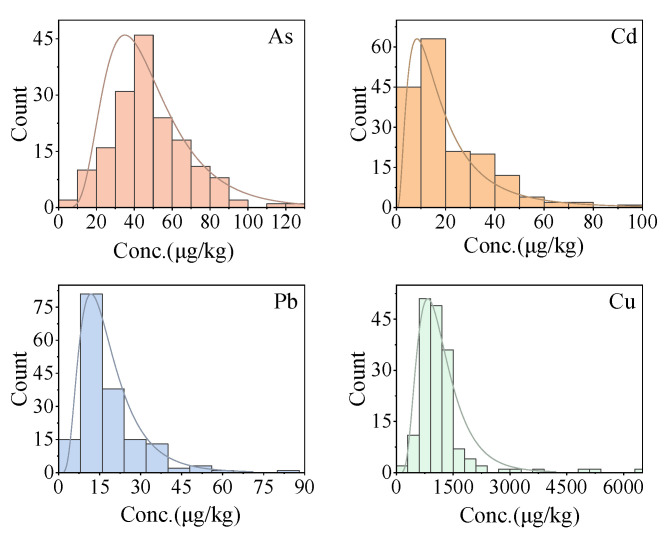
Histogram of As, Cd, Pb, and Cu concentrations in rice.

The concentrations of Cd, As, Pb, and Cu in commercial white rice samples from Nepal are presented in Table 1 and Figure 2. The maximum allowable concentration (MAC) of HMs in white rice established by FAO/WHO [48] were used for comparison with this study. The concentrations of Cd, As, Pb, and Cu in rice from Nepal all showed lognormal distributions (Figure 2). The levels of these HMs in rice from Nepalese markets were compared with those from other countries worldwide (Table S3).

#### 3.1.1. Cadmium

Overall, the geometric mean concentration of Cd in the rice samples was 15.5 ± 16.0 μg/kg (range: 1.31–95.4 μg/kg) (Table 1). The Cd concentrations in all the rice samples were below the MAC for Cd (100 μg/kg) in polished rice recommended by FAO/WHO [48], suggesting that Cd was at a safe level in all the rice samples studied. The lowest geometric mean concentration of Cd (9.93 ± 6.71 μg/kg, range: 1.31–23.5 μg/kg) was in rice collected from Gandaki. The mean Cd concentrations in the rice samples from Madhesh, Lumbini, and Sudurpashchim were all slightly more than 17.0 μg/kg; those from Province 1, Bagmati, and Karnali had mean values close to 15 μg/kg. The mean Cd concentrations in rice were lower in this study than in previous studies in Nepal (50 μg/kg) [49], Sri Lanka (80 μg/kg) [50], and Malaysia (160 μg/kg) [31] and were consistent with those in India (19.1 μg/kg) and Thailand (13.0 μg/kg) [51]. Overall, the concentrations of Cd in this study were low, which may be attributable to the weak effects of some relevant pollution activities, including mining, irrigation, and application of chemical fertilizer and pesticides [52].

#### 3.1.2. Arsenic

The geometric mean concentration of As was 43.4 ± 19.6 μg/kg (range: 6.43–121 μg/kg) (Table 1), which was lower than the MAC of 200 μg/kg [48]. Among the different provinces, the As concentration in Gandaki was highest (54.4 ± 20.3 μg/kg), and that in Sudurpashchim was lowest (35.1 ± 13.9 μg/kg). The high concentration of As in the rice samples collected from Gandaki maybe was related to farming land contaminated by irrigation water with elevated arsenic [30].The As concentrations in the present study were similar to those observed in Sri Lanka (mean 43 μg/kg; range: 2.5–213 μg/kg) [53], but higher than those in Iran (33.5 μg/kg) [54]. However, the mean As concentrations were lower than those in the Nawalparasi district of Terai, Nepal (180 µg/kg), India (79–103 μg/kg) [55], Malaysia (91 μg/kg) [49], and China (119 μg/kg) [38]. Although the mean As concentrations in this study were lower than MAC, soil contamination with As, seriously contaminated irrigation water, and the accumulation in plants have been found in Nepal [30]. Therefore, the exposure of rice to As cannot be ignored in future work.

#### 3.1.3. Lead

The geometric mean concentration of Pb in rice was 16.0 ± 14.0 μg/kg (range: 5.49–121 μg/kg), which was lower than the MAC of 200 μg/kg for rice [48], indicating a safe level of Pb in rice. Among the different provinces, the mean Pb concentrations were highest in rice from Gandaki (19.7 ± 25.5 μg/kg; range: 7.9–87.4 μg/kg) and lowest in rice from Sudurpashchim (12.9 ± 5.94 μg/kg; range: 8.0–26.8 μg/kg). The Pb concentrations were similar to those observed in Sri Lanka (20 ± 1.5 μg/kg; range: 3–61 μg/kg) [56], and much lower than those in Iran (328 ± 81 μg/kg; range: 132–463 μg/kg) [54] and Thailand (419 μg/kg) [51].

#### 3.1.4. Copper

The geometric mean concentration of Cu in rice samples was 1066 ± 1210 μg/kg, with a range of 264–10,059 μg/kg, and only a sample from Dhankuta in eastern Province 1 exceeded the permissible limit of 10,000 μg/kg [48]. Among the provinces, the highest geometric mean concentration of Cu occurred in rice from Madhesh (1364 ± 1601 μg/kg; range: 452–6560 μg/kg), and the lowest was in Lumbini (949 ± 358 μg/kg; range: 271–2016 μg/kg). In the present study, the Cu concentrations of rice in Nepal were similar to those in Thailand (range: 1510–3340 μg/kg) and India (1000 μg/kg), but higher than those in Malaysia (740 μg/kg) [18].

### 3.2. Human Exposure to HMs

The average EDIs of Cu, As, Cd, and Pb were 6.35 ± 3.47, 0.250 ± 0.104, 0.108 ± 0.0888, and 0.100 ± 0.0720 μg/kg bw/day (Table 2 and Table S4), respectively. The average EDIs for the studied elements were all below their RfDs, which are 0.3, 1, 3.5, and 40 μg/kg/day for As, Cd, Pb, and Cu, respectively [42], indicating that exposure to the individual elements during rice consumption is safe in Nepal. The maximal EDI values for Cd and Pb were below their RfDs, but the maximal EDI values for Cu and As were both higher than their corresponding RfDs, and that of As was twice its RfD value. Specifically, the P75th percentile EDI of As was higher than the RfD, demonstrating the high level of As exposure in Nepal.

**Figure 3:**
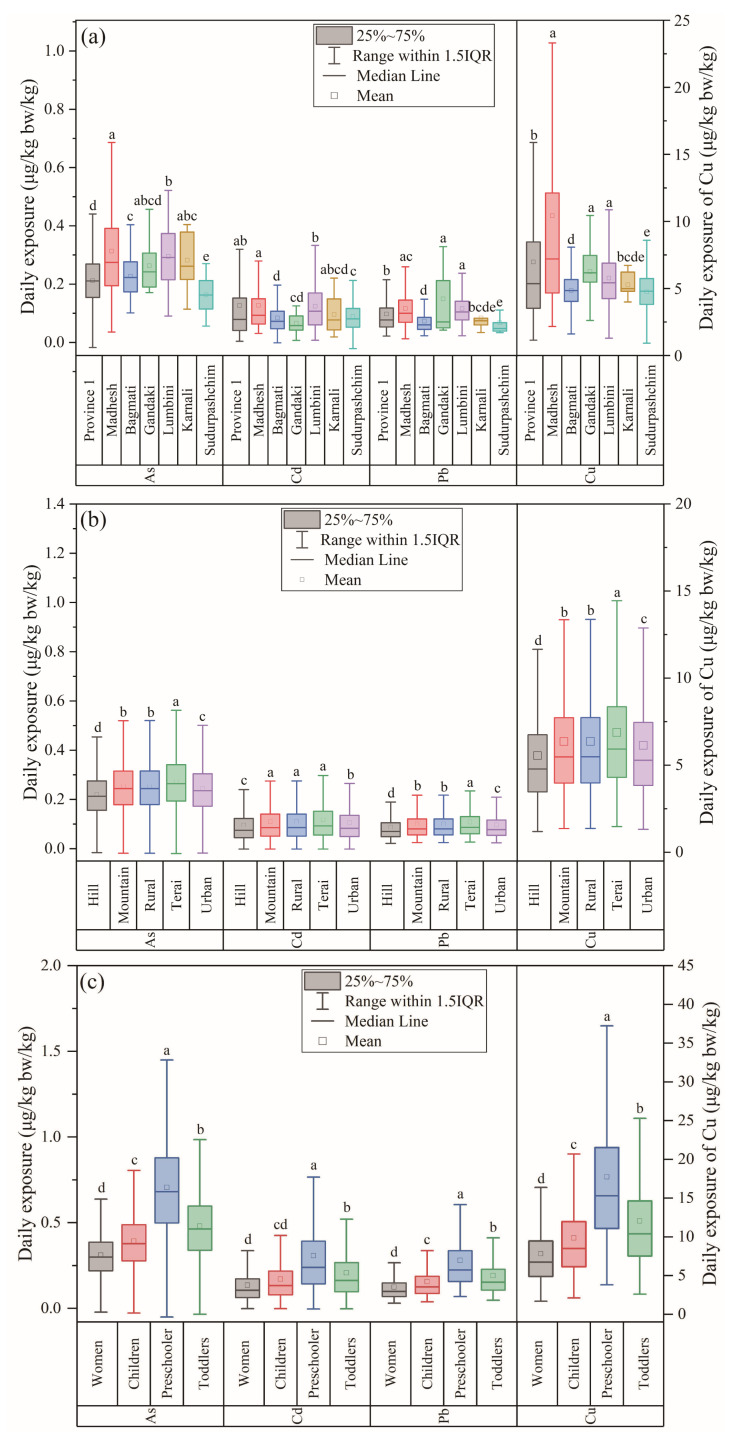
(**a**) EDI distribution of As, Pb, Cd, and Cu in different provinces, (**b**) EDI distribution of As, Pb, Cd, and Cu in different regions, and (**c**) EDI distribution of As, Pb, Cd, and Cu for vulnerable populations in Nepal. Note: different lower case letters suggest the significant difference at level *p* < 0.05.

When the different provinces were compared, ANOVA showed that the highest average EDIs of As, Cd, and Cu were in Madhesh (Figure 3a), with average values of 0.313 ± 0.169, 0.127 ± 0.113, and 10.4 ± 10.7 μg/kg bw/day, respectively. The highest Pb exposure was in Gandaki (0.149 ± 0.136 μg/kg bw/day), but the lowest exposure to As, Pb, and Cu was in Sudurpashchim. The lowest exposure to Cd was recorded in Gandaki (0.0655 ± 0.0358 μg/kg bw/day). The average EDIs of the different elements in all the provinces were below their corresponding RfDs. However, the P95 EDIs of As were above the RfD for As in all the provinces. The P99.9 values for Cu and Cd were above their RfDs in Province 1 and Madhesh, but that for Cu was three times greater than the RfD in Madhesh, and that for Cd was approximately two times greater in Province 1. Therefore, rice consumption poses a potential risk of exposure to Cu and Cd for the residents of Province 1 and Madhesh.

In the different regions, the highest mean EDIs for Cd, As, Pb, and Cu were detected in Terai (0.119, 0.274, 0.108, and 6.88 μg/kg bw/day, respectively), and the lowest mean was in Hill. The P99.9 EDIs of As in all regions were above its RfD. Exposure to HMs was in the order Terai > Rural > Mountain > Urban > Hill for all elements (Figure 3b). Because all rice samples were collected from markets and few from home and we could not identify the sampling sites by region, the national average concentrations were used in this study. In this way, all the differences in exposure risk were caused by the ratio of intake rate to bodyweight. Therefore, the differences in the HM exposure risks reflect the differences in the dietary structures in the different regions.

Because vulnerable populations are more sensitive to HM exposure, women and children were specifically classified according to age to evaluate their exposure to HMs via rice consumption (Table S4). Among the vulnerable populations, preschoolers showed the highest average EDIs for As, Cd, Cu, and Pb (0.705, 0.306, 17.7, and 0.279 µg/kg bw/day, respectively), followed by toddlers, and women were lowest (Figure 3c). Overall, given the higher values for the IR/BW ratio in children, their exposure to HMs was higher than that of women, indicating that the children were more vulnerable to HM exposure in rice. This phenomenon is similar to that seen in methylmercury exposure via rice consumption in Nepal [3]. The average EDIs for As were higher than the RfD in all the vulnerable populations. For Cu and Cd, P99.9 of the EDIs in children, preschoolers, and toddlers were higher than their RfDs, and the P99.9 EDIs for preschoolers were nearly twice the RfDs, indicating a potential exposure risk for these vulnerable populations in Nepal.

### 3.3. Risk Assessment

#### 3.3.1. Non-Carcinogenic Risk (NCR)

The average HQs of Cd, As, Pb, and Cu were all <1, with values of 0.108, 0.834, 0.0282, and 0.159, respectively, in the order As > Cu > Cd > Pb. However, P75–P99.9 of HQ for As was >1 (Table 2 and Table S4). The average HI was 1.13, and P50–P99.9 of HI was in the range of 1.09–2.52, indicating non-carcinogenic adverse health effects. In general, As was the dominant contributor to NCR from HM, accounting for 75.0% of HI, which was similar to that in Thailand (49.3–66.67%) [19].

**Figure 4:**
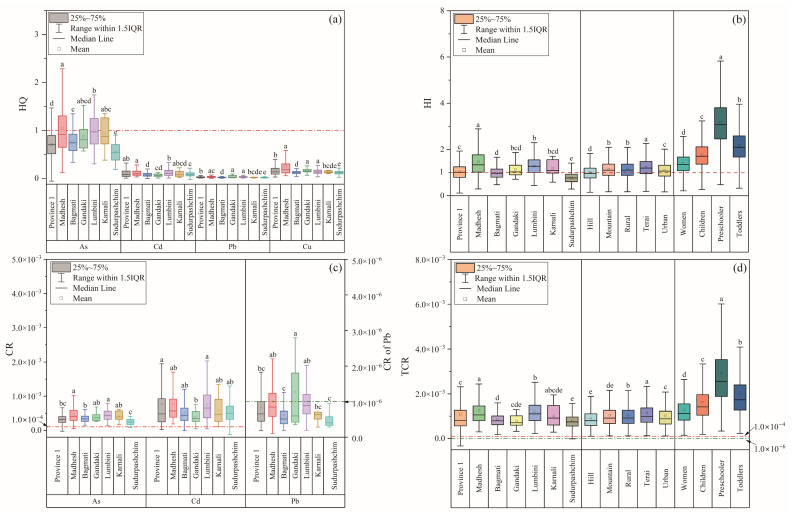
(**a**) The distribution of HQ, (**b**) the distribution of HI, (**c**) the distribution of CR, and (**d**) the distribution of NCR in Nepal. Note: different lower case letters suggest the significant difference at level *p* < 0.05.

The mean HQ for As in Madhesh was >1. Approximately 15.33% of As HQs > 1 were observed in Province 1, 6.78% were in Madhesh, and 3.16% were in Lumbini (Figure 4a), indicating the dominant role of As in NCR. Among the provinces, the order of non-carcinogenic risk was Madhesh > Lumbini > Karnali > Gandaki > Province 1 > Bagamti > Sudurpashchim (Figure 4b). There are diet differences in various provinces, and the intake rate (IR) of rice is highest in Madhesh (Table S2). This is maybe the cause for why the mean HQ of As was higher than other provinces with being above 1.

In different regions, the mean HQ for As was in the range of 0.736–0.912, being close to 1, and the average HIs in all regions were >1, except for Hill, which HI was close to 1, indicating that all regions had NCR. Based on the average HQs of the studied elements and their HIs, the risk of NCR descended in the order Terai > Rural > Mountain > Urban > Hill (Figure S1a–d and Figure 4b).

In terms of the vulnerable population categories, the HQ for As was >1 for all vulnerable populations (women, children, preschoolers, and toddlers), and the values for Cd and Cu were 0.306 and 0.443, respectively, for preschoolers. The potential NCR for vulnerable populations was in the order preschoolers > toddlers > children > women (Figure S1e–h and Figure 4b).

#### 3.3.2. Carcinogenic Risk (CR)

The mean CRs of As and Cd were 3.75 × 10^−4^ and 6.62 × 10^−4^, respectively, which were up to three and six times higher, respectively, than the acceptable limit of 1.0 × 10^−4^. The CRs of As and Cd for P50–P99.9 were in the range of 3.58 × 10^−4^ to 1.01 × 10^−3^ and 5.16 × 10^−4^ to 4.39 × 10^−3^, respectively (Table S5), indicating that As and Cd exposure via rice consumption posed a cancer risk. The average CR of Pb was 8.54 × 10^−7^ (range 2.44 × 10^−7^ to 5.38 × 10^−6^), which was within the acceptable range. The mean TCR was 1.04 × 10^−3^, which exceeded the accepted range of 1.0 × 10^−6^ to 1.0 × 10^−4^. Moreover, the P99.9 of TCR was 4.79 × 10^−3^. The CRs of Cd and As accounted for 63.7% and 36.1% of TCR, respectively, so Cd was the dominant contributor to CR.

The mean CRs for Cd and As in all provinces were above the threshold value of 1.0 × 10^−4^. The highest average CRs for Cd (7.76 × 10^−4^) and As (4.70 × 10^−4^) were both detected in Madhesh, and the lowest CRs of Cd and As were observed in Gandaki (4.40 × 10^−4^) and Lumbini (2.45 × 10^−4^), respectively (Figure 4c, Table S5). The mean TCRs in the different provinces were in the range of 7.88 × 10^−4^ to 1.25 × 10^−3^, in the order Madhesh > Lumbini > Province 1 > Karnali > Bagmati > Gandaki > Sudurpashchim (Figure 4d).

In different regions, the mean CRs of As and Cd were about 3–4 times and 5–7 times higher, respectively, than the limit of 1.0 × 10^−4^ (Figure S2a–c, Table S5). The TCR values in various regions were much higher than 1.0 × 10^−4^, with the highest being in Terai, followed by Rural, Mount, Urban, and Hill (Figure 4d). As mentioned above, these differences were attributable to differences in diet structure.

In the different vulnerable populations, preschoolers had the highest mean CRs for As (1.06 × 10^−3^) and Cd (1.87 × 10^−3^), whereas women had the lowest CRs of 4.65 × 10^−4^ (As) and 8.02 × 10^−4^ (Cd) (Figure S2d–f, Table S5). The mean TCR for women and the younger age groups were all >1.0 × 10^−3^, in the order preschoolers > toddlers > children > women (Figure 4d), implying that all vulnerable populations were exposed to an unacceptable CR. 

## 4. Conclusions

Overall, the concentrations of Cd, As, Pb, and Cu in rice were almost below the maximum allowable concentrations recommended by FAO/WHO, indicating their safety. The average EDIs of all the HMs studied were lower than the RfDs set by USEPA. Among all studied elements, the EDI of As was highest. The highest EDIs for As, Cd, and Cu were observed in Madhesh, and the highest EDI for Pb in Lumbini. Pre-schoolers may be exposed to high levels of HMs when consuming rice. The average HQs of As in Madhesh for women and young age groups were all >1, and the mean HI was also >1, indicating that the consumption of rice posed a potential NCR. Arsenic contributed most of the NCR. The TCR indicated a potential cancer risk among the local inhabitants, which Cd and As contributed to 63.7% and 36.1%, respectively. Future efforts should be made to determine the mechanisms influencing the accumulation of HMs in Nepal. Moreover, in future studies, a blood survey of HMs exposure should be conducted in order to further understand the health effects of HMs via rice ingestion.

## Figures and Tables

**Table 1:** Concentrations of Cd, As, Pb, and Cu in commercial rice samples (n = 170) from each province of Nepal (μg/kg).

Samples Area	Mean ± SD (μg/kg) (Minimum–Maximum)			
	Cd	As	Pb	Cu
Province 1	15.4 ± 21.9	36.6 ± 18.8	15.7 ± 17.3	1078 ± 1920
	(1.76–95.4)	(6.43–92.3)	(5.49–121.4)	(264–10,059)
Madhesh	17.5 ± 13.5	47.9 ± 28.5	17.3 ± 10.3	1364 ± 1601
	(6.80–46.4)	(17.0–121)	(5.72–36.9)	(453–6559)
Bagmati	14.9 ± 11.5	46.4 ± 14.8	13.5 ± 9.21	1000 ± 251
	(2.68–55.9)	(25.3–81.7)	(5.91–43.0)	(519–1556)
Gandaki	9.93 ± 6.71	47.2 ± 15.8	19.7 ± 25.5	1100 ± 398
	(1.31–23.5)	(32.1–85.6)	(7.94–87.4)	(491–1959)
Lumbini	17.2 ± 14.8	49.2 ± 18.1	18.4 ± 10.2	949 ± 358
	(1.73–68.6)	(23.1–85.8)	(6.02–51.3)	(271–2016)
Karnali	15.7 ± 12.5	54.4 ± 20.3	15.6 ± 8.97	1072 ± 194
	(3.86–45.4)	(23.5–83.3)	(6.94–35.1)	(823–1383)
Sudurpashchim	17.0 ± 12.5	35.1 ± 13.9	12.9 ± 5.94	1051 ± 316
	(4.26–51.9)	(17.3–60.2)	(7.98–26.8)	(550–1624)
Total Nepal	15.5 ± 16.0	43.4 ± 19.6	16.0 ± 14.0	1066 ± 1210
	(1.31–95.4)	(6.43–121)	(5.49–121.4)	(264–10,059)
FAO/WHO *	100	200	200	10,000

* The permissible limit of Cd, As, Pb, and Cu [48].

**Table 2:** The estimated EDIs (μg/kg bw/day), HQs, and HIs for exposure to Cd, As, Pb, and Cu via rice consumption in Nepal.

Element	Parameter	Average	P50	P75	P90	P95	P97.5	P99	P99.9
Cd	EDI	0.108	0.0846	0.138	0.212	0.274	0.345	0.440	0.719
	HQ	0.108	0.0846	0.138	0.212	0.274	0.345	0.440	0.719
As	EDI	0.25	0.239	0.313	0.388	0.436	0.484	0.543	0.671
	HQ	0.834	0.795	1.04	1.29	1.45	1.61	1.81	2.24
Pb	EDI	0.100	0.0799	0.120	0.179	0.231	0.292	0.382	0.656
	HQ	0.0282	0.0846	0.138	0.212	0.274	0.345	0.440	0.719
Cu	EDI	6.35	5.51	7.76	10.659	13.0	15.3	18.6	27.7
	HQ	0.159	0.138	0.194	0.266	0.324	0.382	0.466	0.692
	HI	1.13	1.09	1.35	1.62	1.79	1.94	2.17	2.52

## Data Availability

Not applicable.

## Supplementary Materials

The following supporting information can be downloaded at: https://www.mdpi.com/article/10.3390/ijerph20054134/s1, Figure S1: Probability distribution of hazard quotients (HQs) of As (a), Cd (b), Pb (c), and Cu (d) in different regions, and probability distribution of hazard quotients (HQs) of As (e), Cd (f), Pb (g), and Cu (h) for various vulnerable populations; Figure S2: Probability distributions for Carcino-genic risk (CR) of As (a), Cd (b), and Pb (c) in different regions, and probability distributions for Carcinogenic risk (CR) of As (d), Cd (e), and Pb (f) for various vulnerable populations; Table S1: Parameters used in exposure risk assessment in this study; Table S2: Body weight, intake rate, and age for calculating EDI in Nepal; Table S3: Concentrations of Cd, As, Pb, and Cu in market rice reported from other countries; Table S4: Heavy metals exposure (μg/kg bw/day) via rice con-sumption and corresponding HQs, HI; Table S5: Carcinogenic risk (CR) and total carcinogenic risk (TCR) based on Monte Carlo simulation.

## References

[1] Wuana R.A., Okieimen F.E. (2011). Heavy Metals in Contaminated Soils: A Review of Sources, Chemistry, Risks and Best Available Strategies for Remediation. ISRN Ecol..

[2] Naseri M., Vazirzadeh A., Kazemi R., Zaheri F. (2015). Concentration of some heavy metals in rice types available in Shiraz market and human health risk assessment. Food Chem..

[3] Wang L., Han J., Katuwal H.B., Xia P., Xu X., Feng X., Qiu G. (2021). Occurrence of total mercury and methylmercury in rice: Exposure and health implications in Nepal. Ecotoxicol. Environ. Saf..

[4] Abtahi M., Fakhri Y., Oliveri Conti G., Keramati H., Zandsalimi Y., Bahmani Z., Hosseini Pouya R., Sarkhosh M., Moradi B., Amanidaz N. (2017). Heavy metals (As, Cr, Pb, Cd and Ni) concentrations in rice (*Oryza sativa*) from Iran and associated risk assessment: A systematic review. Toxin Rev..

[5] Al-Saleh I., Abduljabbar M. (2017). Heavy metals (lead, cadmium, methylmercury, arsenic) in commonly imported rice grains (*Oryza sativa*) sold in Saudi Arabia and their potential health risk. Int. J. Hyg. Environ. Health.

[6] Khan A., Khan S., Khan M.A., Qamar Z., Waqas M. (2015). The uptake and bioaccumulation of heavy metals by food plants, their effects on plants nutrients, and associated health risk: A review. Environ. Sci. Pollut. Res..

[7] Tchounwou P.B., Patlolla A.K., Centeno J.A. (2003). Carcinogenic and systemic health effects associated with arsenic exposure--A critical review. Toxicol Pathol..

[8] Nejabat M., Kahe H., Shirani K., Ghorbannejad P., Hadizadeh F., Karimi G. (2017). Health risk assessment of heavy metals via dietary intake of wheat in Golestan Province, Iran. Hum. Ecol. Risk Assess..

[9] World Health Organization (2015). WHO Estimates of the Global Burden of Foodborne Diseases: Foodborne Disease Burden Epidemiology Reference Group 2007–2015.

[10] Zheng S., Wang Q., Yuan Y., Sun W. (2020). Human health risk assessment of heavy metals in soil and food crops in the Pearl River Delta urban agglomeration of China. Food Chem..

[11] FAOSTAT. http://www.fao.org/faostat/en/#data/QC.

[12] Perera P.A.C.T., Sundarabarathy T.V., Sivananthawerl T., Kodithuwakku S.P., Edirisinghe U. (2016). Arsenic and Cadmium Contamination in Water, Sediments and Fish is a Consequence of Paddy Cultivation: Evidence of River Pollution in Sri Lanka. Achiev Life Sci..

[13] Li L., Feng H., Wei J. (2020). Toxic element (As and Hg) content and health risk assessment of commercially available rice for residents in Beijing based on their dietary consumption. Environ. Sci. Pollut. Res..

[14] Shimbo S., Zhang Z.-W., Watanabe T., Nakatsuka H., Matsuda-Inoguchi N., Higashikawa K., Ikeda M. (2001). Cadmium and lead contents in rice and other cereal products in Japan in 1998–2000. Sci. Total Environ..

[15] Thomas K.V., Bijlsma L., Castiglioni S., Covaci A., Emke E., Grabic R., Hernández F., Karolak S., Kasprzyk-Hordern B., Lindberg R.H. (2012). Comparing illicit drug use in 19 European cities through sewage analysis. Sci. Total Environ..

[16] Wei J., Gao J., Cen K. (2019). Levels of eight heavy metals and health risk assessment considering food consumption by China’s residents based on the 5th China total diet study. Sci. Total Environ..

[17] Zeng F., Wei W., Li M., Huang R., Yang F., Duan Y. (2015). Heavy Metal Contamination in Rice-Producing Soils of Hunan Province, China and Potential Health Risks. Int. J. Environ. Res. Public Health.

[18] Aziz R.A., Rahim S.A., Sahid I., Idris W.M.R. (2015). Speciation and Availability of Heavy Metals On Serpentinized Paddy Soil and Paddy Tissue. Procedia Soc Behav Sci..

[19] Kukusamude C., Sricharoen P., Limchoowong N., Kongsri S. (2021). Heavy metals and probabilistic risk assessment via rice consumption in Thailand. Food Chem..

[20] Proshad R., Kormoker T., Islam M.S., Chandra K. (2019). Potential health risk of heavy metals via consumption of rice and vegetables grown in the industrial areas of Bangladesh. Hum. Ecol. Risk Assess..

[21] Roychowdhury T., Tokunaga H., Fau-Ando M., Ando M. (2003). Survey of arsenic and other heavy metals in food composites and drinking water and estimation of dietary intake by the villagers from an arsenic-affected area of West Bengal, India. Sci. Total Environ..

[22] Fang Y., Sun X., Yang W., Ma N., Xin Z., Fu J., Liu X., Liu M., Mariga A.M., Zhu X. (2014). Concentrations and health risks of lead, cadmium, arsenic, and mercury in rice and edible mushrooms in China. Food Chem..

[23] Djahed B., Kermani M., Farzadkia M., Taghavi M., Norzaee S. (2020). Exposure to heavy metal contamination and probabilistic health risk assessment using Monte Carlo simulation: A study in the Southeast Iran. J. Environ. Health Sci. Eng..

[24] USEPA (2011). Exposure Factors Handbook.

[25] Abbasi A.M., Iqbal J., Khan M.A., Shah M.H. (2013). Health risk assessment and multivariate apportionment of trace metals in wild leafy vegetables from Lesser Himalayas, Pakistan. Ecotoxicol. Environ. Saf..

[26] Brtnicky M., Pecina V., Hladky J., Radziemska M., Koudelkova Z., Klimanek M., Richtera L., Adamcova D., Elbl J., Galiova M.V. (2019). Assessment of phytotoxicity, environmental and health risks of historical urban park soils. Chemosphere.

[27] USEPA (2001). Risk Assessment Guidance for Superfund, Process for Conducting Probabilistic Risk Assessment.

[28] Hu X.F., Lowe M., Chan H.M. (2021). Mercury exposure, cardiovascular disease, and mortality: A systematic review and dose-response meta-analysis. Environ. Res..

[29] Kayastha S.P. (2014). Heavy metal pollution of agricultural soils and vegetables of bhaktapur district, Nepal. Sci. World.

[30] Dahal B.M., Fuerhacker M., Mentler A., Karki K.B., Shrestha R.R., Blum W.E. (2008). Arsenic contamination of soils and agricultural plants through irrigation water in Nepal. Environ. Pollut..

[31] Meharg A.A., Norton G., Deacon C., Williams P., Adomako E.E., Price A., Zhu Y., Li G., Zhao F.J., McGrath S. (2013). Variation in rice cadmium related to human exposure. Environ. Sci. Technol..

[32] Fillman T., Shimizu-Furusawa H., Ng C.F.S., Parajuli R.P., Watanabe C. (2016). Association of cadmium and arsenic exposure with salivary telomere length in adolescents in Terai, Nepal. Environ. Res..

[33] Maharjan M., Watanabe C., Ahmad S.A., Ohtsuka R. (2005). Arsenic contamination in drinking water and skin manifestations in lowland Nepal: The first community-based survey. Am. J. Trop. Med. Hyg..

[34] Gumma M.K., Gauchan D., Nelson A., Pandey S., Rala A. (2011). Temporal changes in rice-growing area and their impact on livelihood over a decade: A case study of Nepal. Agric. Ecosyst. Environ..

[35] Central Bureau of Statistics (CBS) (2016). Statistical Year Book.

[36] Ministry of Finance (MoF) (2014). Economic Survey.

[37] Li D., Zhang Q., Sun D., Yang C., Luo G. (2022). Accumulation and risk assessment of heavy metals in rice: A case study for five areas of Guizhou Province, China. Environ. Sci. Pollut. Res..

[38] Qian Y., Chen C., Zhang Q., Li Y., Chen Z., Li M. (2010). Concentrations of cadmium, lead, mercury and arsenic in Chinese market milled rice and associated population health risk. Food Control..

[39] Panter-Brick C. (1992). Women’s work and child nutrition: The food intake of 0–4 year old children in rural Nepal. Ecol. Food Nutr..

[40] Saville N.M., Maharjan M., Manandhar D.S., Harris-Fry H.A. (2020). Equity implications of rice fortification: A modelling study from Nepal. Public Health Nutr..

[41] Ren Y., Lin M., Liu Q., Zhang Z., Fei X., Xiao R., Lv X. (2021). Contamination assessment, health risk evaluation, and source identification of heavy metals in the soil-rice system of typical agricultural regions on the southeast coast of China. Environ. Sci. Pollut. Res..

[42] USEPA (2000). Risk-Based Concentration Table.

[43] Zheng N., Wang Q., Zhang X., Zheng D., Zhang Z., Zhang S. (2007). Population health risk due to dietary intake of heavy metals in the industrial area of Huludao City, China. Sci. Total Environ..

[44] USEPA Integrated Risk Information System (IRIS)-Cd Chemical Assessment Summary, National Center for Environmental Assessment. https://cfpub.epa.gov/ncea/iris2/chemicalLanding.cfm?substance_nmbr=141.

[45] USEPA (2019). Guidelines for Human Exposure Assessment.

[46] Ferreira-Baptista L., De Miguel E. (2005). Geochemistry and risk assessment of street dust in Luanda, Angola: A tropical urban environment. Atmos. Environ..

[47] USEPA (United States Environmental Protection Agency) Risk Assessment Guidance for Superfund (RAGS), Volume I, Human Health Evaluation Manual (Part E, Supplemental Guidance for Dermal Risk Assessment). https://www.epa.gov/risk/risk-assessment-guidancesuperfund-rags-part-e.

[48] Codex Alimentarius Commission (2011). Joint FAO/WHO Food Standards Programme Codex Committee on Contaminants in Foods.

[49] Praveena S.M., Omar N.A. (2017). Heavy metal exposure from cooked rice grain ingestion and its potential health risks to humans from total and bioavailable forms analysis. Food Chem..

[50] Liu L., Han J., Xu X., Xu Z., Abeysinghe K.S., Atapattu A.J., De Silva P., Lu Q., Qiu G. (2020). Dietary exposure assessment of cadmium, arsenic, and lead in market rice from Sri Lanka. Environ. Sci. Pollut. Res..

[51] Rahman M.A., Rahman M.M., Reichman S.M., Lim R.P., Naidu R. (2014). Heavy metals in Australian grown and imported rice and vegetables on sale in Australia: Health hazard. Ecotoxicol. Environ. Saf..

[52] Zakaria Z., Zulkafflee N.S., Mohd Redzuan N.A., Selamat J., Ismail M.R., Praveena S.M., Tóth G., Abdull Razis A.F. (2021). Understanding Potential Heavy Metal Contamination, Absorption, Translocation and Accumulation in Rice and Human Health Risks. Plants.

[53] Diyabalanage S., Fonseka S., Dasanayake D., Chandrajith R. (2017). Environmental exposures of trace elements assessed using keratinized matrices from patients with chronic kidney diseases of uncertain etiology (CKDu) in Sri Lanka. J. Trace Elem. Med Biol..

[54] Morekian R., Mirlohi M., Azadbakht L., Maracy M.R. (2013). Heavy metal distribution frequency in Iranian and imported rice varieties marketed in central Iran, Yazd, 2012. Int. J. Environ. Health Eng..

[55] Jallad K.N. (2015). Heavy metal exposure from ingesting rice and its related potential hazardous health risks to humans. Environ. Sci. Pollut. Res..

[56] Norton G.J., Williams P.N., Adomako E.E., Price A.H., Zhu Y., Zhao F.J., McGrath S., Deacon C.M., Villada A., Sommella A. (2014). Lead in rice: Analysis of baseline lead levels in market and field collected rice grains. Sci. Total Environ..

