# Supplementary material for: Human Dietary Exposure to Heavy Metals via Rice in Nepal
Source: Int J Environ Res Public Health. 2023 Feb 25;20(5):4134. doi: 10.3390/ijerph20054134 (PMC10001872; doi:10.3390/ijerph20054134)
Supplement: Supplementary file 1 [file ijerph-20-04134-s001.zip › ijerph-2169049-supplementary - new.pdf]

Human Dietary Exposure to Heavy Metals Via Rice in Nepal

Yuxiao Shao [1,2], Xiaohang Xu [2,3], Le Wang [2], Jialiang Han [2,4], Hem Bahadur Katuwal [5], Shulin Jiao [1,*] and Guangle Qiu [2]

[1] School of Geography and Environmental Science, Guizhou Normal University, Guiyang 550025, China; shaoyuxiao29@163.com
[2] State Key Laboratory of Environmental Geochemistry, Institute of Geochemistry, Chinese Academy of Sciences, Guiyang 550081, China; xxh1119@foxmail.com (X.X.); wangle0602@foxmail.com (L.W.); hanjialiang@mail.gyig.ac.cn (J.H.); qiuguangle@vip.skleg.cn (G.Q.)
[3] Key Laboratory of Karst Georesources and Environment, Ministry of Education, College of Resources and Environmental Engineering, Guizhou University, Guiyang 550025, China
[4] University of Chinese Academy of Sciences, Beijing 100049, China
[5] Center for Integrative Conservation, Xishuangbanna Tropical Botanical Garden, Chinese Academy of Sciences, Mengla 666303, China; hembdrkatuwal@gmail.com
* Correspondence: jiaoshulin@gznu.edu.cn

Table of Contents

Table S1. Parameters used in exposure risk assessment in this study.
Table S2. Body weight, intake rate, and age for calculating EDI in Nepal.
Table S3. Concentrations of Cd, As, Pb, and Cu in market rice reported from other countries.
Table S4. Heavy metals exposure (μg/kg bw/day) via rice consumption and corresponding HQs, HI.
Table S5. Carcinogenic risk (CR) and total carcinogenic risk (TCR) based on Monte Carlo simulation.
Figure S1. Probability distribution of hazard quotients (HQs) of As (a), Cd (b), Pb (c), and Cu (d) in different regions, and probability distribution of hazard quotients (HQs) of As (e), Cd (f), Pb (g), and Cu (h) for various vulnerable populations.
Figure S2. Probability distributions for Carcinogenic risk (CR) of As (a), Cd (b), and Pb (c) in different regions, and probability distributions for Carcinogenic risk (CR) of As (d), Cd (e), and Pb (f) for various vulnerable populations.

Table S1. Parameters used in exposure risk assessment in this study.

Parameter	Description	Values	Unit	Reference
CF	Concentration of the metal in rice		μg/kg	This study
EF	Exposure Frequency	365	day/year	[1]
IR	Rice Intake Rate		g/day	[2]
ED	Exposure Duration	70	years	[1]
BW	Body weight		kg	[3]
AT	Average exposure time	365× ED (non-carcinogenic)<br>365× 70 (carcinogenic)	day	[4]

Table S2. Body weight, intake rate, and age for calculating EDI in Nepal.

Category	Body weight (kg)	Intake rate (g/day)	Age
Province 1	60	308.3	Adult
Madhesh	60	345.7	Adult
Bagmati	60	280.3	Adult
Gandaki	60	320.0	Adult
Lumbini	60	339.2	Adult
Karnail	60	291.5	Adult
Sudurpashchim	60	259.8	Adult
Terai	60	341.5	Adult
Hill	60	275.6	Adult
Mountain	60	315.8	Adult
Urban	60	304.5	Adult
Rural	60	316.2	Adult
Women	50	322.4	15-49 years
Children	32	260.6	5-12 years
Toddlers	8.63	86.0	13-24 months
Preschooler	12	176.0	37-50 months
Total Nepal	60	313.0	Adult

Table S3. Concentrations of Cd, As, Pb, and Cu in market rice reported from other countries.

Rice origin	Sampling/ purchasing site	Sample size	Cd (µg/kg)	Range (µg/kg)	As (µg/kg)	Range (µg/kg)	Pb (µg/kg)	Range (µg/kg)	Cu (µg/kg)	Range (µg/kg)	Reference
Nepal	7 provinces	170	15.3 ± 16.1	9.00-95.4	43.4±19.6	6.4-120.8	16± 14	5.5-121.4	1065.5±1210.3	236.6-10058.5	This study
Nepal	Nepal	12	50	13.9-80	No	No	No	No	No	No	[5]
Nepal	Nepal	12	No	No	No	No	33.0±26.0	3.0-71	No	No	[6]
Sri Lanka	Sri Lanka	165	80±130	3-727	77±40	19-217	31±50	1.0-345	No	No	[7]
Sri Lanka	Sri Lanka	226	85	No	43	2.50-213	No	No	No	No	[8]
Sri Lanka	Sri Lanka	41	No	No	No	No	20±1.5	3.0-61.0	No	No	[6]
Ghana	Ghana	428	20	5.00-270	No	No	No	No	No	No	[5]
Malaysia	Malaysia	66	95	No	91	No	110	No	2400	No	[9]
India	India	64	No	No	No	No	23.0 ± 40.0	3.0-266	No	No	[6]
Iran	Yazd, Iran	108	37.3 ± 23.4	No	33.5 ± 18.4	No	328 ± 44.8	132 -463	No	No	[10]
Spain	Spain	89	No	No	No	No	15.0 ± 11.0	3.0-89.0	No	No	[6]
China	20 provinces	712	50	ND-740	119	ND-490	62	ND-400	No	No	[1]
Brazil	Brazil	23	No	No	No	No	5.30 ± 4.10	0.4-14.5	No	No	[11]
China	22 provinces	92	80.0 ± 70.0	ND-373	50.0 ± 10.0	ND-310	100-140	ND-524	No	No	[12]
Korea	Korea	63	21	10-32	122	43-199	No	No	1850	1290-2530	[13]
Bangladesh	Bangladesh	6	73	69-77	No	No	19	5-28	1600	1300-1900	[14]
Pakistan	Pakistan	9	4.5	ND-8.9	No	No	67	3-168	2600	1900-3600	[14]
Thailand	Thailand	12	13	6.3-17.8	No	No	419	96-921	3500	2000-6800	[14]
India	India	15	19.1	ND-36.4	No	No	267	2-637	5900	2300-11800	[14]
Australia	Australia	21	7.5	8.7-17.1	No	No	375	16-1248	2900	1000-9400	[14]
Italy	Italy	6	0.5	0.2-0.8	No	No	317	44-841	5800	1300-13000	[14]
Vietnam	Vietnam	3	No	No	No	No	No	No	9300	7900-10700	[5]
Iran	Iran	36	33.7 ± 39	1-222	369± 94	91-872	123 ±140	5-574	3095±439420	1690-4590	[15]
India	India	17	No	No	No	No	No	No	5900	2300-11800	[5]

Table S4. Heavy metals exposure (μg/kg bw/day) via rice consumption and corresponding HQs, HI.

Element	Categories	Mean±Std	Min	Max	P50	P75	P90	P95	P97.5	P99	P99.9
Cd	Province 1	0.127±0.161	0.00904	0.490	0.0789	0.152	0.276	0.379	0.511	0.760	1.78
	Madhesh	0.127±0.113	0.0392	0.267	0.0930	0.150	0.241	0.326	0.427	0.565	1.07
	Bagmati	0.0841±0.0540	0.0125	0.261	0.0723	0.107	0.151	0.183	0.219	0.270	0.418
	Lumbini	0.124±0.0830	0.00977	0.388	0.107	0.170	0.241	0.287	0.330	0.371	0.457
	Sudurpashchim	0.0889±0.0523	0.0184	0.225	0.0808	0.116	0.155	0.184	0.213	0.256	0.349
	Terai	0.119±0.0970	0.00746	0.543	0.0925	0.152	0.235	0.301	0.371	0.488	0.773
	Hill	0.0958±0.0783	0.00602	0.438	0.0746	0.123	0.189	0.243	0.299	0.394	0.623
	Mountain	0.110±0.0897	0.00690	0.502	0.0855	0.141	0.217	0.278	0.343	0.451	0.714
	Urban	0.106±0.0865	0.00665	0.484	0.0824	0.135	0.209	0.268	0.331	0.435	0.689
	Rural	0.110±0.0898	0.00691	0.503	0.0856	0.141	0.217	0.278	0.343	0.452	0.715
	Women	0.134±0.110	0.00845	0.615	0.105	0.172	0.266	0.341	0.420	0.553	0.875
	Children	0.170±0.139	0.0107	0.777	0.132	0.217	0.335	0.430	0.530	0.698	1.11
	Toddlers	0.208±0.170	0.0131	0.950	0.162	0.266	0.411	0.527	0.649	0.854	1.35
	Preschooler	0.306±0.250	0.0192	1.399	0.238	0.392	0.604	0.775	0.955	1.26	1.99
	Total Nepal	0.108±0.0888	0.00684	0.498	0.0846	0.138	0.212	0.274	0.345	0.440	0.719
	HQ	0.108±0.0888	0.00684	0.498	0.0846	0.138	0.212	0.274	0.345	0.440	0.719
As	Province 1	0.211±0.095	0.0330	0.474	0.211	0.269	0.325	0.364	0.402	0.453	0.600
	Madhesh	0.313±0.169	0.0980	0.696	0.275	0.391	0.529	0.632	0.748	0.896	1.28
	Bagmati	0.227±0.0670	0.118	0.382	0.223	0.277	0.321	0.345	0.362	0.377	0.395
	Lumbini	0.295±0.101	0.131	0.485	0.291	0.374	0.435	0.464	0.483	0.498	0.513
	Sudurpashchim	0.163±0.0582	0.0751	0.261	0.163	0.212	0.245	0.256	0.263	0.267	0.270
	Terai	0.274±0.112	0.0366	0.688	0.264	0.341	0.418	0.476	0.532	0.587	0.730
	Hill	0.221±0.0905	0.0295	0.555	0.213	0.275	0.338	0.384	0.429	0.474	0.589
	Mountain	0.253±0.104	0.0338	0.636	0.244	0.315	0.387	0.440	0.492	0.543	0.675
	Urban	0.244±0.100	0.0326	0.613	0.235	0.304	0.373	0.424	0.474	0.523	0.651
	Rural	0.253±0.104	0.0339	0.637	0.244	0.316	0.387	0.440	0.493	0.543	0.676
	Women	0.310±0.127	0.0414	0.779	0.299	0.386	0.474	0.539	0.603	0.665	0.827
	Children	0.392±0.160	0.0523	0.98	0.378	0.488	0.598	0.681	0.761	0.840	1.04
	Toddlers	0.479±0.196	0.0641	1.20	0.462	0.597	0.732	0.833	0.931	1.03	1.28
	Preschooler	0.705±0.289	0.0943	1.77	0.680	0.879	1.078	1.23	1.37	1.51	1.88
	Total Nepal	0.250±0.104	0.0335	0.630	0.239	0.313	0.388	0.436	0.484	0.543	0.671
	HQ	0.834±0.346	0.112	2.10	0.795	1.04	1.29	1.45	1.61	1.81	2.24
Pb	Province 1	0.0977±0.0710	0.0282	0.624	0.077	0.118	0.180	0.230	0.291	0.364	0.631
	Madhesh	0.117±0.0693	0.0330	0.213	0.100	0.145	0.205	0.249	0.295	0.352	0.534
	Bagmati	0.0732±0.0479	0.0276	0.201	0.0602	0.0860	0.124	0.158	0.197	0.248	0.451
	Lumbini	0.116±0.0551	0.0340	0.290	0.104	0.141	0.187	0.221	0.253	0.298	0.404
	Sudurpashchim	0.0680±0.0925	0.0346	0.116	0.0476	0.0677	0.109	0.157	0.226	0.336	1.03
	Terai	0.108±0.0769	0.0313	0.691	0.0867	0.130	0.193	0.247	0.306	0.407	0.755
	Hill	0.0875±0.0621	0.0252	0.558	0.0700	0.105	0.156	0.200	0.247	0.329	0.610
	Mountain	0.100±0.0712	0.0289	0.639	0.0802	0.121	0.179	0.229	0.283	0.377	0.699
	Urban	0.0967±0.0686	0.0279	0.616	0.0773	0.116	0.172	0.220	0.273	0.363	0.673
	Rural	0.100±0.0712	0.0289	0.64	0.0803	0.121	0.179	0.229	0.284	0.377	0.699
	Women	0.123±0.0871	0.0354	0.783	0.0982	0.148	0.219	0.280	0.347	0.461	0.856
	Children	0.155±0.110	0.0447	0.989	0.124	0.187	0.277	0.354	0.438	0.582	1.08
	Toddlers	0.190±0.135	0.0547	1.21	0.152	0.228	0.338	0.433	0.537	0.713	1.32
	Preschooler	0.279±0.198	0.0806	1.78	0.223	0.336	0.498	0.637	0.790	1.05	1.95
	Total Nepal	0.100±0.0720	0.0287	0.633	0.0799	0.120	0.179	0.231	0.292	0.382	0.656
	HQ	0.0282±0.0202	0.00705	0.269	0.0846	0.138	0.212	0.274	0.345	0.440	0.719
Cu	Province 1	6.98±5.60	1.35	51.7	5.36	8.47	13.00	17.0	22.2	29.6	47.7
	Madhesh	10.4±10.7	2.61	37.8	7.20	12.1	19.96	28.6	38.0	51.2	120
	Bagmati	4.85±1.17	2.43	7.27	4.85	5.66	6.370	6.76	7.09	7.49	8.33
	Lumbini	5.77±2.17	1.53	11.4	5.42	6.89	8.540	9.87	11.1	12.5	16.5
	Sudurpashchim	4.75±1.38	2.38	7.03	4.80	5.75	6.507	6.96	7.33	7.68	8.50
	Terai	6.88±3.82	1.50	57.3	5.93	8.36	11.498	14.2	17.0	20.0	30.9
	Hill	5.55±3.08	1.21	46.2	4.78	6.74	9.28	11.5	13.7	16.2	24.9
	Mountain	6.36±3.53	1.39	52.9	5.48	7.73	10.633	13.1	15.7	18.5	28.5
	Urban	6.13±3.41	1.34	51.0	5.28	7.45	10.251	12.7	15.2	17.9	27.5
	Rural	6.37±3.54	1.39	53.0	5.49	7.74	10.644	13.2	15.7	18.5	28.6
	Women	7.79±4.33	1.70	64.8	6.71	9.47	13.022	16.1	19.3	22.7	34.9
	Children	9.84±5.47	2.15	81.9	8.48	12.0	16.450	20.3	24.3	28.7	44.1

	Toddlers	12.0±6.69	2.63	100.2	10.37	14.6	20.129	24.9	29.8	35.1	54.0
	Preschooler	17.7±9.85	3.87	147.5	15.27	21.5	29.625	36.6	43.8	51.6	79.5
	Total Nepal	6.35±3.47	1.38	52.5	5.51	7.76	10.659	13.0	15.3	18.6	27.7
	HQ	0.159±0.0867	0.0344	1.31	0.138	0.194	0.266	0.324	0.382	0.466	0.692
	HI	1.13±0.368	0.240	2.49	1.09	1.35	1.62	1.79	1.94	2.17	2.52

Table S5. Carcinogenic risk (CR) and total carcinogenic risk (TCR) based on Monte Carlo simulation.

Element	Categories	Mean±Std	P50	P75	P90	P95	P97.5	P99	P99.9
	Province 1	$7.73\times10^{-4}\pm9.80\times10^{-4}$	4.81×10^{-4}	9.29×10^{-4}	1.69×10^{-3}	2.31×10^{-3}	3.12×10^{-3}	4.64×10^{-3}	1.09×10^{-2}
	Madhesh	$7.76\times10^{-4}\pm6.89\times10^{-4}$	5.67×10^{-4}	9.13×10^{-4}	1.47×10^{-3}	1.99×10^{-3}	2.60×10^{-3}	3.45×10^{-3}	6.55×10^{-3}
	Bagmati	$5.13\times10^{-4}\pm3.03\times10^{-4}$	4.41×10^{-4}	6.51×10^{-4}	9.21×10^{-4}	1.11×10^{-3}	1.33×10^{-3}	1.65×10^{-3}	2.55×10^{-3}
	Lumbini	$7.58\times10^{-4}\pm5.06\times10^{-4}$	6.53×10^{-4}	1.03×10^{-3}	1.47×10^{-3}	1.75×10^{-3}	2.01×10^{-3}	2.26×10^{-3}	2.79×10^{-3}
	Sudurpashchim	$5.42\times10^{-4}\pm3.19\times10^{-4}$	4.93×10^{-4}	7.11×10^{-4}	9.48×10^{-4}	1.12×10^{-3}	1.30×10^{-3}	1.56×10^{-3}	2.13×10^{-3}
	Terai	$7.24\times10^{-4}\pm5.92\times10^{-4}$	5.64×10^{-4}	9.27×10^{-4}	1.43×10^{-3}	1.83×10^{-3}	2.26×10^{-3}	2.98×10^{-3}	4.71×10^{-3}
Cd	Hill	$5.84\times10^{-4}\pm4.78\times10^{-4}$	4.55×10^{-4}	7.48×10^{-4}	1.15×10^{-3}	1.48×10^{-3}	1.83×10^{-3}	2.40×10^{-3}	3.80×10^{-3}
	Mountain	$6.70\times10^{-4}\pm5.47\times10^{-4}$	5.22×10^{-4}	8.57×10^{-4}	1.32×10^{-3}	1.70×10^{-3}	2.09×10^{-3}	2.75×10^{-3}	4.36×10^{-3}
	Urban	$6.46\times10^{-4}\pm5.28\times10^{-4}$	5.03×10^{-4}	8.26×10^{-4}	1.28×10^{-3}	1.64×10^{-3}	2.02×10^{-3}	2.65×10^{-3}	4.20×10^{-3}
	Rural	$6.70\times10^{-4}\pm5.48\times10^{-4}$	5.22×10^{-4}	8.58×10^{-4}	1.32×10^{-3}	1.70×10^{-3}	2.09×10^{-3}	2.76×10^{-3}	4.36×10^{-3}
	Women	$8.20\times10^{-4}\pm6.70\times10^{-4}$	6.39×10^{-4}	1.05×10^{-3}	1.62×10^{-3}	2.08×10^{-3}	2.56×10^{-3}	3.37×10^{-3}	5.34×10^{-3}
	Children	$1.04\times10^{-3}\pm8.47\times10^{-4}$	8.07×10^{-4}	1.33×10^{-3}	2.05×10^{-3}	2.63×10^{-3}	3.24×10^{-3}	4.26×10^{-3}	6.74×10^{-3}
	Toddlers	$1.27\times10^{-3}\pm1.04\times10^{-4}$	9.87×10^{-4}	1.62×10^{-3}	2.50×10^{-3}	3.21×10^{-3}	3.96×10^{-3}	5.21E-03	8.25×10^{-3}
	Preschooler	$1.87\times10^{-3}\pm1.53\times10^{-3}$	1.45×10^{-3}	2.39×10^{-3}	3.69×10^{-3}	4.73×10^{-3}	5.83×10^{-3}	7.67×10^{-3}	1.21×10^{-3}
	Total Nepal	$6.62\times10^{-4}\pm5.42\times10^{-4}$	5.16×10^{-4}	8.39×10^{-4}	1.29×10^{-3}	1.67×10^{-3}	2.10×10^{-3}	2.68×10^{-3}	4.39×10^{-3}
	Province 1	$3.17\times10^{-4}\pm1.42\times10^{-4}$	3.16×10^{-4}	4.03×10^{-4}	4.87×10^{-4}	5.46×10^{-4}	6.03×10^{-4}	6.80×10^{-4}	9.00×10^{-4}
	Madhesh	$4.70\times10^{-4}\pm2.54\times10^{-4}$	4.12×10^{-4}	5.87×10^{-4}	7.94×10^{-4}	9.47×10^{-4}	1.12×10^{-3}	1.34×10^{-3}	1.91×10^{-3}
	Bagmati	$3.41\times10^{-4}\pm1.00\times10^{-4}$	3.34×10^{-4}	4.15×10^{-4}	4.82×10^{-4}	5.18×10^{-4}	5.43×10^{-4}	5.66×10^{-4}	5.92×10^{-4}
	Lumbini	$4.43\times10^{-4}\pm1.51\times10^{-4}$	4.37×10^{-4}	5.60×10^{-4}	6.52×10^{-4}	6.96×10^{-4}	7.24×10^{-4}	7.47×10^{-4}	7.70×10^{-4}
	Sudurpashchim	$2.45\times10^{-4}\pm8.74\times10^{-5}$	2.44×10^{-4}	3.18×10^{-4}	3.67×10^{-4}	3.84×10^{-4}	3.94×10^{-4}	4.00×10^{-4}	4.05×10^{-4}
	Terai	$4.11\times10^{-4}\pm1.68\times10^{-4}$	3.96×10^{-4}	5.11×10^{-4}	6.27×10^{-4}	7.13×10^{-4}	7.98×10^{-4}	8.80×10^{-4}	1.09×10^{-3}
As	Hill	$3.31\times10^{-4}\pm1.36\times10^{-4}$	3.20×10^{-4}	4.13×10^{-4}	5.06×10^{-4}	5.76×10^{-4}	6.44×10^{-4}	7.10×10^{-4}	8.84×10^{-4}
	Mountain	$3.80\times10^{-4}\pm1.56\times10^{-4}$	3.66×10^{-4}	4.73×10^{-4}	5.80×10^{-4}	6.60×10^{-4}	7.38×10^{-4}	8.14×10^{-4}	1.01×10^{-3}
	Urban	$3.66\times10^{-4}\pm1.50\times10^{-4}$	3.53×10^{-4}	4.56×10^{-4}	5.59×10^{-4}	6.36×10^{-4}	7.11×10^{-4}	7.85×10^{-4}	9.76×10^{-4}
	Rural	$3.80\times10^{-4}\pm1.56\times10^{-4}$	3.67×10^{-4}	4.73×10^{-4}	5.81×10^{-4}	6.61×10^{-4}	7.39×10^{-4}	8.15×10^{-4}	1.01×10^{-3}
	Women	$4.65\times10^{-4}\pm1.91\times10^{-4}$	4.48×10^{-4}	5.79×10^{-4}	7.11×10^{-4}	8.08×10^{-4}	9.04×10^{-4}	9.97×10^{-4}	1.24×10^{-3}
	Children	$5.87\times10^{-4}\pm2.41\times10^{-4}$	5.66×10^{-4}	7.32×10^{-4}	8.98×10^{-4}	1.02×10^{-3}	1.14×10^{-3}	1.26×10^{-3}	1.57×10^{-3}
	Toddlers	$7.19\times10^{-4}\pm2.94\times10^{-4}$	6.93×10^{-4}	8.95×10^{-4}	1.10×10^{-3}	1.25×10^{-3}	1.40×10^{-3}	1.54×10^{-3}	1.92×10^{-3}
	Preschooler	$1.06\times10^{-3}\pm4.33\times10^{-4}$	1.02×10^{-3}	1.32×10^{-3}	1.62×10^{-3}	1.84×10^{-3}	2.06×10^{-3}	2.27×10^{-3}	2.82×10^{-3}
	Total Nepal	$3.75\times10^{-4}\pm1.55\times10^{-4}$	3.58×10^{-4}	4.70×10^{-4}	5.82×10^{-4}	6.53×10^{-4}	7.27×10^{-4}	8.14×10^{-4}	1.01×10^{-3}
	Province 1	$8.30\times10^{-7}\pm6.04\times10^{-7}$	6.55×10^{-7}	1.00×10^{-6}	1.53×10^{-6}	1.95×10^{-6}	2.47×10^{-6}	3.10×10^{-6}	5.36×10^{-6}
	Madhesh	$9.92\times10^{-7}\pm5.89\times10^{-7}$	8.49×10^{-7}	1.23×10^{-6}	1.74×10^{-6}	2.11×10^{-6}	2.51×10^{-6}	2.99×10^{-6}	4.54×10^{-6}
	Bagmati	$6.22\times10^{-7}\pm4.07\times10^{-7}$	5.11×10^{-7}	7.31×10^{-7}	1.05×10^{-6}	1.34×10^{-6}	1.68×10^{-6}	2.11×10^{-6}	3.83×10^{-6}
	Lumbini	$9.86\times10^{-7}\pm4.68\times10^{-7}$	8.88×10^{-7}	1.20×10^{-6}	1.59×10^{-6}	1.88×10^{-6}	2.15×10^{-6}	2.53×10^{-6}	3.43×10^{-6}
	Sudurpashchim	$5.78\times10^{-7}\pm7.87\times10^{-7}$	4.05×10^{-7}	5.75×10^{-7}	9.23×10^{-7}	1.33×10^{-6}	1.92×10^{-6}	2.86×10^{-6}	8.75×10^{-6}
	Terai	$9.22\times10^{-7}\pm6.54\times10^{-7}$	7.37×10^{-7}	1.11×10^{-6}	1.64×10^{-6}	2.10×10^{-6}	2.60×10^{-6}	3.46×10^{-6}	6.42×10^{-6}
Pb	Hill	$7.44\times10^{-7}\pm5.28\times10^{-7}$	5.95×10^{-7}	8.94×10^{-7}	1.33×10^{-6}	1.70×10^{-6}	2.10×10^{-6}	2.79×10^{-6}	5.18×10^{-6}
	Mountain	$8.53\times10^{-7}\pm6.05\times10^{-7}$	6.82×10^{-7}	1.02×10^{-6}	1.52×10^{-6}	1.94×10^{-6}	2.41×10^{-6}	3.20×10^{-6}	5.94×10^{-6}
	Urban	$8.22\times10^{-7}\pm5.83\times10^{-7}$	6.57×10^{-7}	9.88×10^{-7}	1.47×10^{-6}	1.87×10^{-6}	2.32×10^{-6}	3.09×10^{-6}	5.72×10^{-6}
	Rural	$8.54\times10^{-7}\pm6.05\times10^{-7}$	6.82×10^{-7}	1.03×10^{-6}	1.52×10^{-6}	1.95×10^{-6}	2.41×10^{-6}	3.20×10^{-6}	5.94×10^{-6}
	Women	$1.04\times10^{-6}\pm7.41\times10^{-7}$	8.35×10^{-7}	1.26×10^{-6}	1.86×10^{-6}	2.38×10^{-6}	2.95×10^{-6}	3.92×10^{-6}	7.27×10^{-6}
	Children	$1.32\times10^{-6}\pm9.36\times10^{-7}$	1.05×10^{-6}	1.59×10^{-6}	2.35×10^{-6}	3.01×10^{-6}	3.73×10^{-6}	4.95×10^{-6}	9.19×10^{-6}
	Toddlers	$1.61\times10^{-6}\pm1.14\times10^{-6}$	1.29×10^{-6}	1.94×10^{-6}	2.88×10^{-6}	3.68×10^{-6}	4.56×10^{-6}	6.06×10^{-6}	1.12×10^{-5}
	Preschooler	$2.38\times10^{-6}\pm1.69\times10^{-6}$	1.90×10^{-6}	2.86×10^{-6}	4.23×10^{-6}	5.42×10^{-6}	6.71×10^{-6}	8.92×10^{-6}	1.65×10^{-5}
	Total Nepal	$8.54\times10^{-7}\pm6.12\times10^{-7}$	6.79×10^{-7}	1.02×10^{-6}	1.52×10^{-6}	1.96×10^{-6}	2.48×10^{-6}	3.25×10^{-6}	5.57×10^{-6}
	TCR	$1.04\times10^{-3}\pm5.63\times10^{-4}$	9.06×10^{-4}	1.25×10^{-3}	1.70×10^{-3}	2.09×10^{-3}	2.49×10^{-3}	3.08×10^{-3}	4.79×10^{-3}

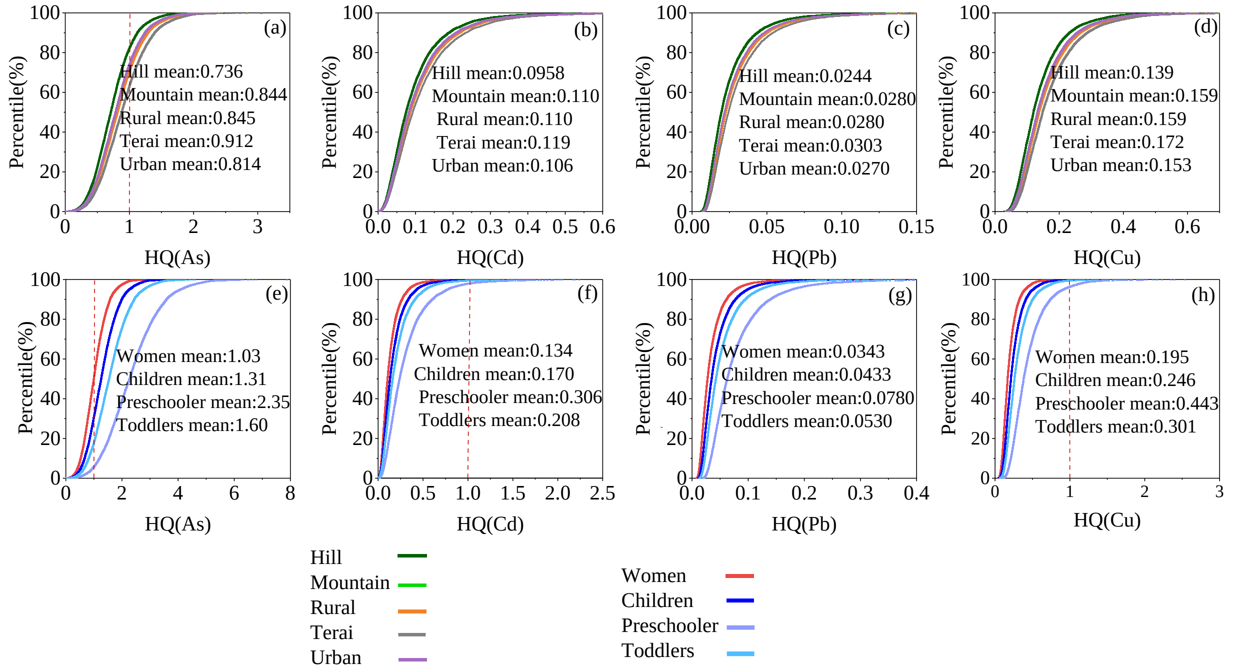

Figure S1. Probability distribution of hazard quotients (HQs) of As (a), Cd (b), Pb (c), and Cu (d) in different regions, and probability distribution of hazard quotients (HQs) of As (e), Cd (f), Pb (g), and Cu (h) for various vulnerable populations.

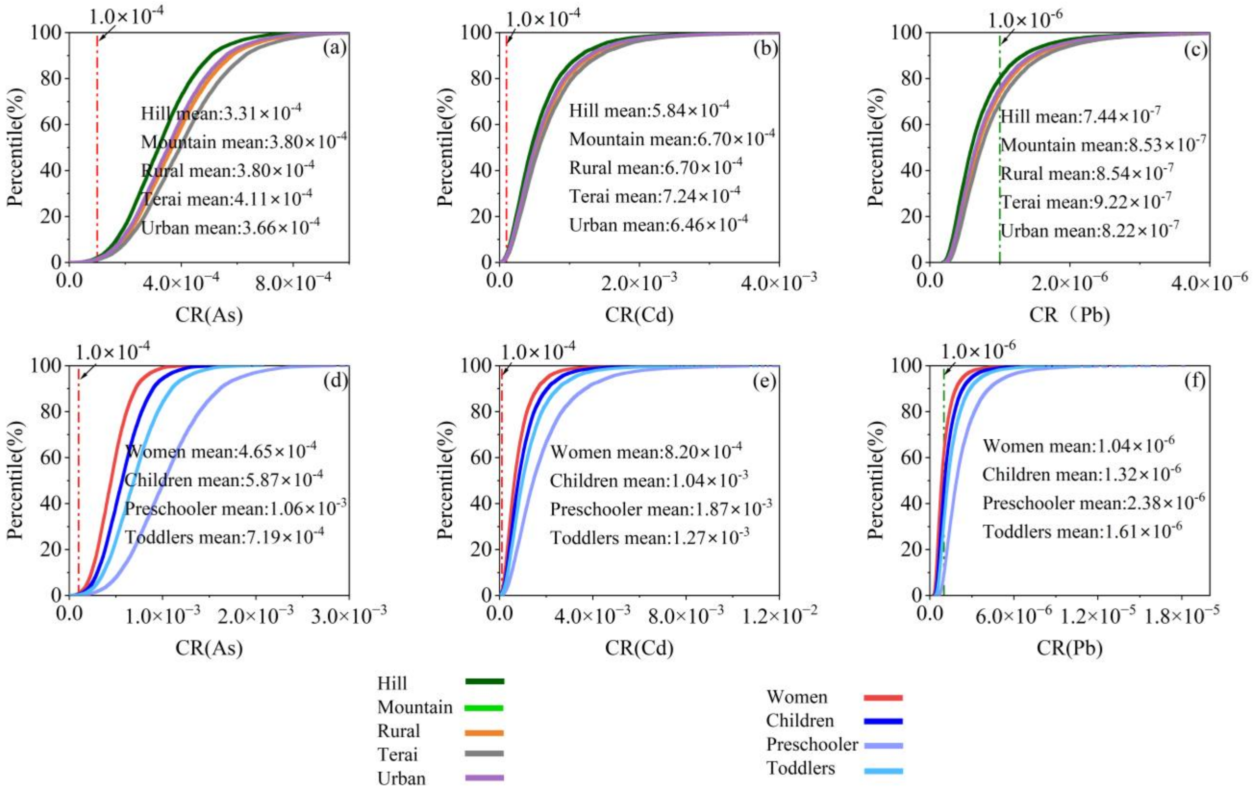

Figure S2. Probability distributions for Carcinogenic risk (CR) of As (a), Cd (b), and Pb (c) in different regions, and probability distributions for Carcinogenic risk (CR) of As (d), Cd (e), and Pb (f) for various vulnerable populations.

References

1. Qian, Y.; Chen, C.; Zhang, Q.; Li, Y.; Chen, Z.; Li, M. Concentrations of cadmium, lead, mercury and arsenic in Chinese market milled rice and associated population health risk. *Food Control* **2010,** *21* (12), 1757-1763.
2. Saville, N. M.; Maharjan, M.; Manandhar, D. S.; Harris-Fry, H. A. Equity implications of rice fortification: a modelling study from Nepal. *Public Health Nutr.* **2020,** *23* (15), 2824-2839.
3. Panter-Brick; Catherine. Women's work and child nutrition: The food intake of 0–4 year old children in rural Nepal. *Ecol. Food Nutr.* **1992,** *29* (1), 207-230.
4. MEP. Exposure Factors Handbook of Chinese Population (Adults). Beijing: Ministry of 175 Environmental Protection, Chinese. 2013.

5. Meharg, A. A.; Norton, G.; Deacon, C.; Williams, P.; Adomako, E. E.; Price, A.; Zhu, Y.; Li, G.; Zhao, F. J.; McGrath, S.; Villada, A.; Sommella, A.; De Silva, P. M.; Brammer, H.; Dasgupta, T.; Islam, M. R. Variation in rice cadmium related to human exposure. *Environ. Sci. Technol.* **2013,** *47* (11), 5613-8.
6. Norton, G. J.; Williams, P. N.; Adomako, E. E.; Price, A. H.; Zhu, Y.; Zhao, F. J.; McGrath, S.; Deacon, C. M.; Villada, A.; Sommella, A.; Lu, Y.; Ming, L.; De Silva, P.; Brammer, H.; Dasgupta, T.; Islam, M. R.; Meharg, A. A. Lead in rice: analysis of baseline lead levels in market and field collected rice grains. *Sci. Total Environ.* **2014,** *485-486*, 428-434.
7. Liu, L.; Han, J.; Xu, X.; Xu, Z.; Abeysinghe, K. S.; Atapattu, A. J.; De Silva, P.; Lu, Q.; Qiu, G. Dietary exposure assessment of cadmium, arsenic, and lead in market rice from Sri Lanka. *Environ. Sci. Pollut. Res.* **2020,** *27* (34), 42704-42712.
8. Diyabalanage, S.; Fonseka, S.; Dasanayake, D.; Chandrajith, R. Environmental exposures of trace elements assessed using keratinized matrices from patients with chronic kidney diseases of uncertain etiology (CKDu) in Sri Lanka. *J. Trace Elem. Med Biol.* **2017,** *39*, 62-70.
9. Praveena, S. M.; Omar, N. A. Heavy metal exposure from cooked rice grain ingestion and its potential health risks to humans from total and bioavailable forms analysis. *Food Chem.* **2017,** *235*, 203-211.
10. Mirlohi, M.; Morekian, R.; Azadbakht, L.; Maracy, M. Heavy metal distribution frequency in Iranian and imported rice varieties marketed in central Iran, Yazd, 2012. *Int. J. Environ. Health Eng.* **2013,** *2* (1).
11. Batista, B. L.; Nacano, L. R.; Freitas, R. d.; Oliveira-Souza, V. C. d.; Barbosa, F. Determination of Essential (Ca, Fe, I, K, Mo) and Toxic Elements (Hg, Pb) in Brazilian Rice Grains and Estimation of Reference Daily Intake. *Food Nutr. Sci.* **2012,** *03* (01), 129-134.
12. Fang, Y.; Sun, X.; Yang, W.; Ma, N.; Xin, Z.; Fu, J.; Liu, X.; Liu, M.; Mariga, A. M.; Zhu, X.; Hu, Q. Concentrations and health risks of lead, cadmium, arsenic, and mercury in rice and edible mushrooms in China. *Food Chem.* **2014,** *147*, 147-51.
13. Jung, M. C.; Yun, S. T.; Lee, J. S.; Lee, J. U. Baseline study on essential and trace elements in polished rice from South Korea. *Environ Geochem Health* **2005,** *27* (5-6), 455-64.
14. Rahman, M. A.; Rahman, M. M.; Reichman, S. M.; Lim, R. P.; Naidu, R. Heavy metals in Australian grown and imported rice and vegetables on sale in Australia: health hazard. *Ecotoxicol. Environ. Saf.* **2014,** *100*, 53-60.
15. Djahed, B.; Taghavi, M.; Farzadkia, M.; Norzaee, S.; Miri, M. Stochastic exposure and health risk assessment of rice contamination to the heavy metals in the market of Iranshahr, Iran. *Food Chem. Toxicol.* **2018,** *115*, 405-412.
